# Male breast cancer in Northern Tanzania: Clinicopathology and predictors of two-year survival (2016–2022)

**DOI:** 10.1371/journal.pone.0361615

**Published:** 2026-11-06

**Authors:** Timon Stephen Emaely, Alex Mremi, Elizabeth F. Msoka, Rebeca Mutashobya, Blandina T. Mmbaga, Kondo Chilonga

**Affiliations:** 1 Department of General Surgery, KCMC University, Moshi, Tanzania; 2 Department of General Surgery, Kilimanjaro Christian Medical Centre, Moshi, Tanzania; 3 Kilimanjaro Clinical Research Institute, Moshi, Tanzania; 4 Cancer Care Centre, Kilimanjaro Christian Medical Centre, Moshi, Tanzania; 5 Department of Pathology, Kilimanjaro Christian Medical Centre, Moshi, Tanzania; Prashanti Cancer Care Mission, INDIA

## Abstract

**Background:**

Male breast cancer (BC) is rare, accounting to approximately 1% of all breast cancers worldwide. Patients often present with advanced disease, contributing to poor outcomes. This study aimed to estimate two-year overall survival (OS) and identify clinicopathological and health-system–related predictors of survival among men with breast cancer treated at Kilimanjaro Christian Medical Centre (KCMC).

**Methods:**

A retrospective cohort study was conducted among 55 histologically confirmed male BC patients managed at KCMC between 2016 and 2022. The primary outcome was two-year overall survival, defined as time from histological diagnosis to death from any cause or censoring at two years. The dependent variables were clinical and pathological factors and the key independent variable was overall survival (OS). Descriptive statistics summarized participants characteristics. Kaplan–Meier curves, log-rank and Cox proportional hazards regression were used to estimate the OS and the factors associated. A p-value <0.05 was considered statistically significant.

**Results:**

The mean age at diagnosis was 62.2 ± 14.3 years, with most patients (54.6%) aged between 50–70 years. Over half (52.7%) presented with stage III or IV disease, and 21.8% had distant metastasis. Invasive ductal carcinoma (IDC) was the predominant histological subtype (85.5%), and luminal B was the most common molecular profile (23.6%). Surgery was performed in 60% of patients, chemotherapy in 47.3%, hormonal therapy in 30.9%, and radiotherapy in only 9.1%. The overall two-year survival rate was 67.3%. Independent predictors of poor survival included age > 70 years (p = 0.009), stage IV disease (p = 0.005), delayed receipt of histopathological results (> 2 weeks), delayed surgery (>6 weeks), and non-receipt of hormonal therapy or radiotherapy.

**Conclusion:**

Male BC in Northern Tanzania remains a disease for older who frequently present with advanced-stages. Two-year overall survival remains suboptimal and is strongly influenced by advanced disease stage, older age, diagnostic and treatment delays, and limited access to adjuvant therapies. Strengthening early diagnosis, timely pathology reporting, and access to radiotherapy and hormonal therapy may substantially improve outcomes.

## Introduction

Male (BC) is rare, accounting for approximately 1% of all BC and about 1% of cancers occurring in men worldwide [1–3]. Although uncommon, it’s incidence has shown a gradual increase over recent decades, with global estimates indicating an approximate 26% rise [4]. In high-income countries such as the United States,thousands new cases are diagnosed annually, whereas in Sub-Saharan Africa (SSA), the proportional burden appears higher, with some reports suggesting an incidence of up to 15% [1]. The observed regional variation has been linked to multiple factors, including awareness, early detection, and improved diagnostic access are critical interventions for both men and women [5]..

In Tanzania, male BC represents about 6% of all breast cancer cases [6]. Similar to other SSA countries, late presentation, limited awareness, sociocultural beliefs, and the scarcity of specialized oncology services contribute to delayed diagnosis and poorer survival outcomes compared to high-income settings [7]. Importantly, survival data specific to men with breast cancer in Tanzania remain scarce, limiting evidence-based planning of services for this population. Many patients first seek care from traditional healers, leading to prolonged symptom duration before medical evaluation. Consequently, advanced disease (stage III–IV) at presentation is common [8,9].

Despite sharing similarities with female BC, male differ in several important aspects. Men are typically diagnosed 5–10 years later than women, and they exhibit distinct hormonal and genetic profiles, including a higher likelihood of oestrogen receptor positivity and increased prevalence of BRCA2 mutations [10]. The small volume of male breast tissue also facilitates earlier extension into surrounding structures, accelerating disease progression [3]. Histologically, invasive ductal carcinoma predominates, whereas lobular carcinoma is relatively rare due to the absence of breast lobules in men [3].

Although treatment recommendations for men are largely extrapolated from female BC guidelines, growing evidence suggests biological and clinical differences warranting dedicated research [11]. In low-resource settings, limited access to timely diagnostic services, radiotherapy, hormonal therapy, and multidisciplinary care further complicates management and may negatively influence survival [12].

Therefore, the primary objective of this study was to estimate two-year overall survival among men diagnosed with breast cancer at KCMC. Secondary objectives were to describe clinicopathological characteristics and to identify patient-, tumors-, and health-system–related predictors of survival. We hypothesized that advanced stage at diagnosis, older age, and delays in diagnostic and therapeutic processes would be associated with poorer survival [13,14].

## Materials and methods

### Study design and setting

This was a retrospective hospital-based cohort study conducted at KCMC, a tertiary referral and teaching hospital in Northern Tanzania. KCMC provides comprehensive cancer care services, including diagnostic imaging, pathology, surgery, and systemic therapy. Radiotherapy services are not available on-site and require referral to external centres, which may introduce treatment delays.

### Study population

The study included all male patients with histologically confirmed primary BC treated at KCMC between January 2016 and December 2022. Data for this study were collected from August 19, 2024 to October 30, 2024. Patients with secondary breast cancer were excluded. Follow-up information was obtained from the hospital’s cancer registry and patient records.

### Histopathological review

Given the rarity of invasive lobular carcinoma in males, all cases initially diagnosed as invasive lobular carcinoma underwent an independent histopathological review by a board-certified senior anatomical pathologist (co-author) with extensive experience in breast pathology. The original hematoxylin and eosin (H&E) slides were re-examined to verify the diagnosis based on established histomorphological criteria. The diagnoses were confirmed following this independent expert review before inclusion in the final analysis.

### Variables and definitions

The primary outcome variable was two-year OS**,** defined as the time from diagnosis to death from any cause or last documented follow-up within the two-year period. Cause-specific mortality data were not consistently available; therefore, all-cause mortality was used, which may overestimate cancer-related deaths, particularly in older patients.

Independent variables included: sociodemographic factors, clinical characteristics, pathological features, and treatment-related variables. Diagnostic delay was defined as waiting more than two weeks for histopathological results, and treatment delay was defined as surgery performed more than six weeks after diagnosis.

### Data collection and management

Data were collected using a structured extraction tool designed for this study. Patient records, pathology reports, and the electronic hospital management system (EHMS) were reviewed. All information was coded and anonymized to maintain confidentiality prior to analysis. Missing data, particularly for nodal status and molecular subtype, were documented and reported descriptively.

### Statistical analysis

Data were analysed using SPSS version 25. Kaplan–Meier methods estimated two-year overall survival, and log-rank tests compared survival distributions. Variables were included in multivariable Cox regression based on clinical relevance and statistical significance in univariate analysis (p < 0.05). Adjusted hazard ratios (HRs) with 95% confidence intervals (CIs) were reported. Proportional hazards assumptions were assessed graphically and found to be acceptable.Missing data were handled using multiple imputation by chained equations (MICE) under the assumption that data were Missing at Random (MAR).

### Ethical considerations

Ethical approval for this study was granted by the KCMC University Research Ethics Committee on 13^th^, August, 2024, (Ref. No. PG 34/2024). The consent to participate in this study was waived by the ethics committee of our institution. Permission to access patient records was obtained from relevant hospital authorities. Although the authors had access to information that could identify individual participants during or after data collection, however, all data presented in this study were handled confidentially and used solely for research purposes.

## Results

### Sociodemographic characteristics

Among 55 patients with histologically confirmed male breast cancer treated at KCMC between January 2016 and December 2022, the mean age at diagnosis was 62.2 years (SD ± 14.3), with the majority aged 50–70 years (54.6%). Most patients were married (92.7%), predominantly self-employed (70.9%), and over half resided in rural areas (54.6%). Regarding social and clinical risk characteristics, 34.6% reported a history of smoking and 43.6% alcohol use. More than half of patients paid for healthcare out-of-pocket (58.2%), while 41.8% had health insurance coverage. (Refer to Table 1)

**Table 1:** Sociodemographic characteristics of male breast cancer patients (N = 55).

Characteristic	Category	N	%
**Age (years)**	<50	10	18.2
	50–70	30	54.6
	>70	15	27.2
	Mean ± SD	62.2 ± 14.3	
**Marital status**	Single	3	5.5
	Married	51	92.7
	Divorced	1	1.8
**Education level**	Primary/None	19	34.5
	Secondary	20	36.4
	Higher	16	29.1
**Employment**	Employed	11	20.0
	Self-employed	39	70.9
	Unemployed	5	9.1
**Residence**	Rural	30	54.5
	Urban	25	45.5
**Smoking history**	No	36	65.5
	Yes	19	34.5
**Alcohol use**	No	31	56.4
	Yes	24	43.6
**Health insurance**	Insured	23	41.8
	Cash payment	32	58.2

### Clinical characteristics

Among the 55 patients, 67.27% presented with symptoms lasting more than six months before diagnosis, indicating delayed presentation. Most patients had unilateral breast involvement, with the left breast slightly more affected (52.7%). A family history of breast or ovarian cancer was reported in 14.6% of cases, while 47.3% had comorbidities, mainly hypertension and diabetes mellitus. Regarding tumour characteristics, 45.5% had positive lymph node involvement, although nodal status was undocumented in 40% of cases. Most patients presented with advanced disease, with 30.9% diagnosed at stage III and 21.8% at stage IV, while distant metastasis was present in 21.8% of patients. (Refer to Table 2)

**Table 2:** Clinical characteristics at diagnosis (N = 55).

Characteristic	Category	n	%
**Concurrent malignancy**	No	54	98.2
	Yes (prostate)	1	1.8
**Family history of BC/OC**	No	47	85.5
	Yes	8	14.5
**BMI**	Underweight	9	16.4
	Normal	19	34.5
	Overweight	20	36.4
	Obese	7	12.7
**Symptom duration**	≤6 months	18	32.7
	>6 months	37	67.3
**Breast side**	Left	29	52.7
	Right	26	47.3
**Laterality**	Unilateral	55	100
**Comorbidities**	None	29	52.7
	≥1 comorbidity	26	47.3
**Regional nodes**	Negative	8	14.5
	Positive	22	45.5
	Not documented	25	40.0
**TNM stage**	I	10	18.2
	II	16	29.1
	III	17	30.9
	IV	12	21.8
**Distant metastasis**	No	43	78.2
	Yes	12	21.8

### Pathological characteristics

The predominant histological type was invasive ductal carcinoma (85.5%), followed by invasive lobular carcinoma (14.5%). Most patients (81.8%) received their diagnostic results within two weeks. Tumour grading showed that Grade 3 tumours were most common (34.6%), followed by Grade 2 (27.3%). Luminal B was the most frequently identified molecular subtype (23.6%), although molecular classification was unavailable for 45.5% of patients. (Refer to Table 3).

**Table 3:** Pathological characteristics (N = 55).

Characteristic	Category	n	%
**Histological type**	Invasive ductal carcinoma	47	85.5
	Invasive lobular carcinoma	8	14.5
**Time to pathology report**	≤2 weeks	45	81.8
	>2 weeks	10	18.2
**Nottingham grade**	Grade I	8	14.6
	Grade II	15	27.3
	Grade III	19	34.5
	Unknown	13	23.6
**Molecular subtype**	Luminal A	8	14.6
	Luminal B	13	23.6
	HER2-positive	5	9.1
	Triple negative	4	7.3
	Unknown	25	45.4

### Treatment modalities

Among the 55 male breast cancer patients, 60% underwent surgery, mainly modified radical mastectomy (84.9%). Chemotherapy was administered to 47.3%, with most receiving adjuvant therapy (65.4%), while delays in initiation beyond 12 weeks occurred among 58.8% of those receiving adjuvant chemotherapy. Radiotherapy was rarely utilized (18.5%), and all cases received it as adjuvant treatment. Hormonotherapy was received by 30.9% of patients, with the majority initiated promptly after indication. (Table 4)

**Table 4:** Treatment modalities and timelines (N = 55).

Treatment	Category	n	%
**Surgery**	No	22	40.0
	Yes	33	60.0
**Surgical approach** (n = 33)	Lumpectomy	5	15.2
	Modified radical mastectomy	28	84.8
**Time to surgery**	≤6 weeks	21	63.6
	>6 weeks	12	36.4
**Chemotherapy**	No	29	52.7
	Yes	26	47.3
**Chemotherapy intent** (n = 26)	Adjuvant	17	65.4
	Neoadjuvant	9	34.6
**Time to adjuvant CT** (n = 17)	4–12 weeks	7	41.2
	>12 weeks	10	58.8
**Radiotherapy(n = 27)**	No	22	81.5
	Yes (adjuvant)	5	18.5
**Hormonal therapy**	No	38	69.1
	Yes	17	30.9

### Two-year overall survival

Overall survival was calculated from the date of histological diagnosis. During the two-year follow-up period, a total of 18 deaths were recorded, while the remaining patients were censored at last follow-up or at two years. The Kaplan–Meier estimated two-year overall survival rate was 67.3% (95% CI: 0.53–0.78) (Fig 1).

**Fig 1:**
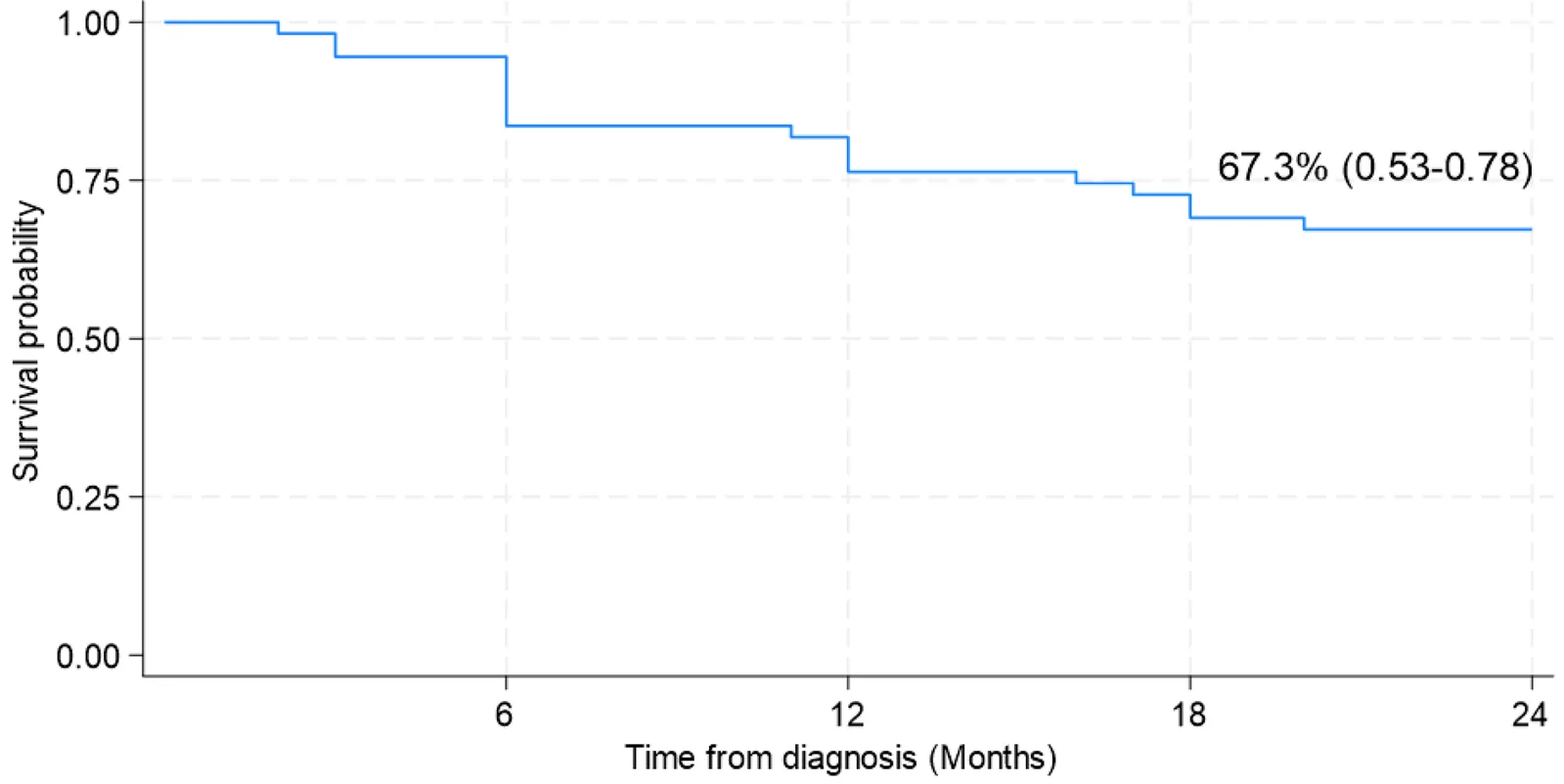
Kaplan-Meier for 2-year overall survival in male breast cancer patient.

### Predictors of Survival

In multivariable Cox regression analysis (Table 5**),** age > 70 years and stage IV disease were independently associated with increased mortality risk (Fig 2, Fig 3**).**Delays in receiving histopathological results (>2 weeks) (Fig 4) and delays in surgery (>6 weeks) (Fig 5) remained significant predictors of poorer survival after adjustment for disease stage.

**Table 5:** Predictors of two-year overall survival (Cox regression).

Variable	Category	Adjusted HR	95% CI	p-value
**Age (years)**	<50	1.00	Reference	
	50–70	1.87	0.13–27.23	0.804
	>70	**5.02**	**2.50–41.16**	**0.009**
**TNM stage**	I	1.00	Reference	
	II	4.08	0.47–33.15	0.074
	III	5.47	0.71–48.57	0.139
	IV	**4.99**	**1.76–59.13**	**0.005**
**Radiotherapy**	No	1.00	Reference	
	Yes	**0.35**	**0.06–0.62**	**0.018**
**Hormonal therapy**	No	1.00	Reference	
	Yes	**0.09**	**0.01–0.99**	**0.049**
**Time to pathology**	≤2 weeks	1.00	Reference	
	>2 weeks	**3.44**	**1.78–11.16**	**0.013**
**Time to surgery**	≤6 weeks	1.00	Reference	
	>6 weeks	**4.15**	**1.07–16.11**	**0.027**

Bold values indicate statistical significance (p < 0.05).

**Fig 2:**
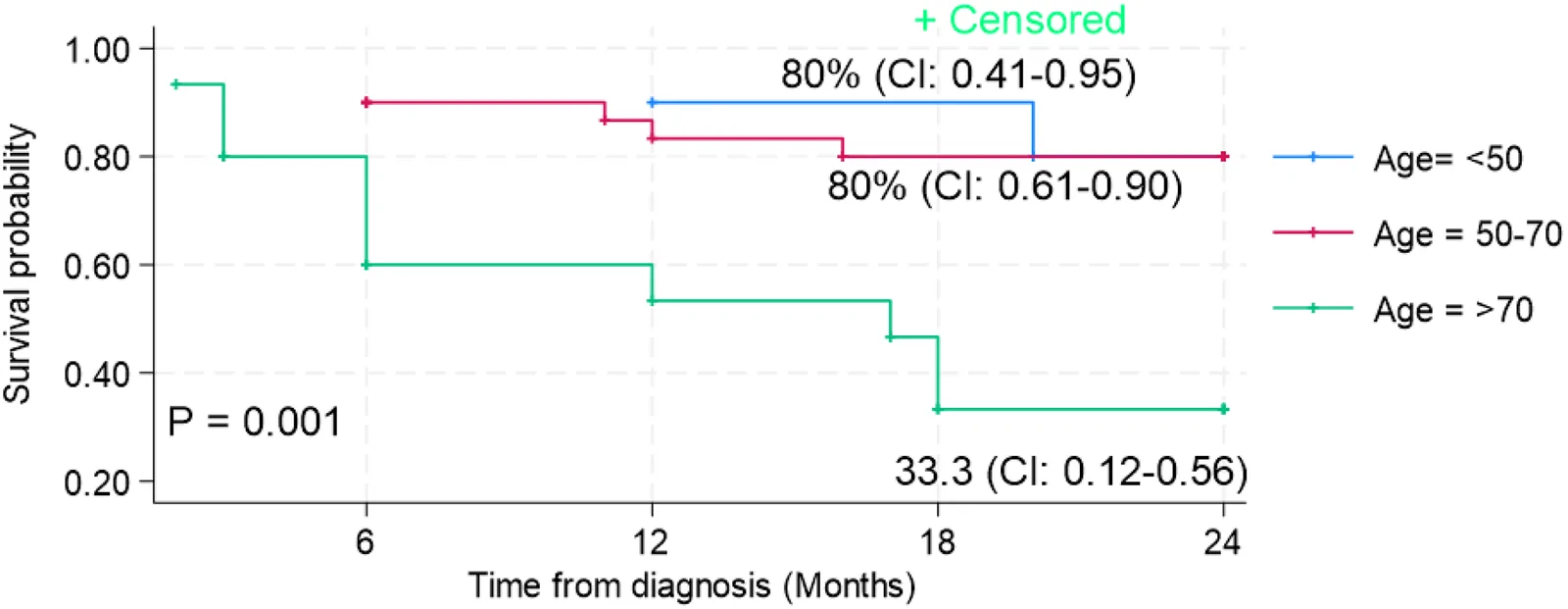
Kaplan-Meier survival estimates by age.

**Fig 3:**
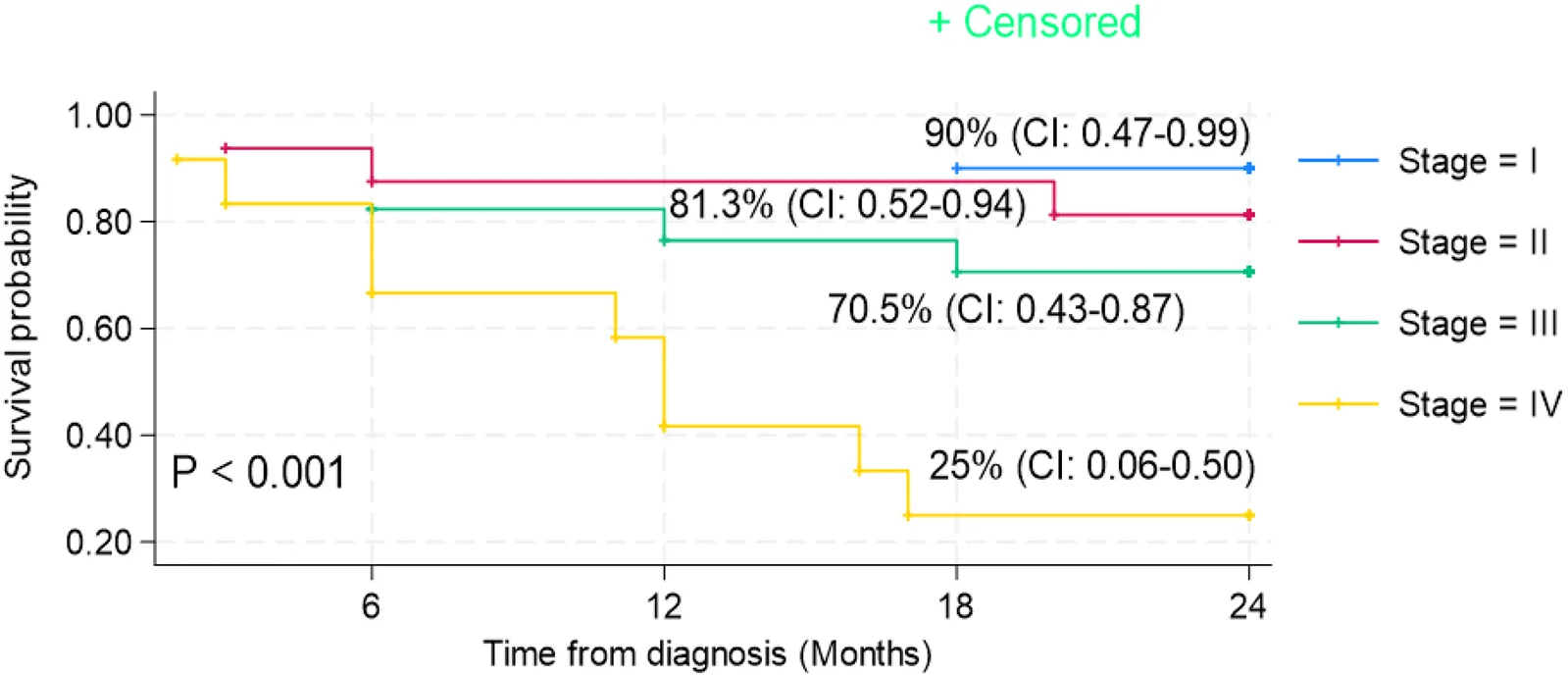
Kaplan-Meier survival estimates by TNM stage.

**Fig 4:**
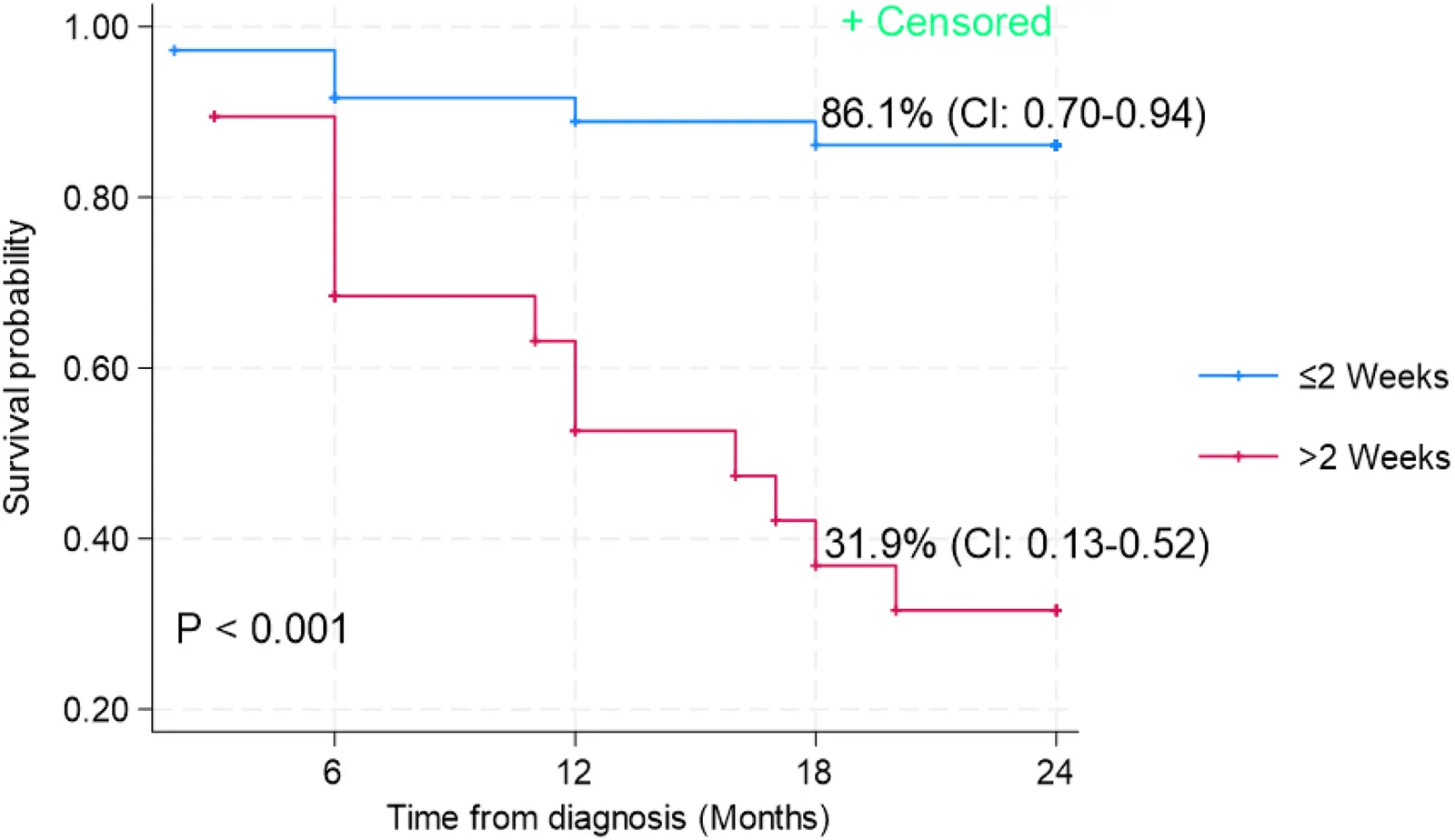
Kaplan-Meier survival estimates by time waiting for biopsy results.

**Fig 5:**
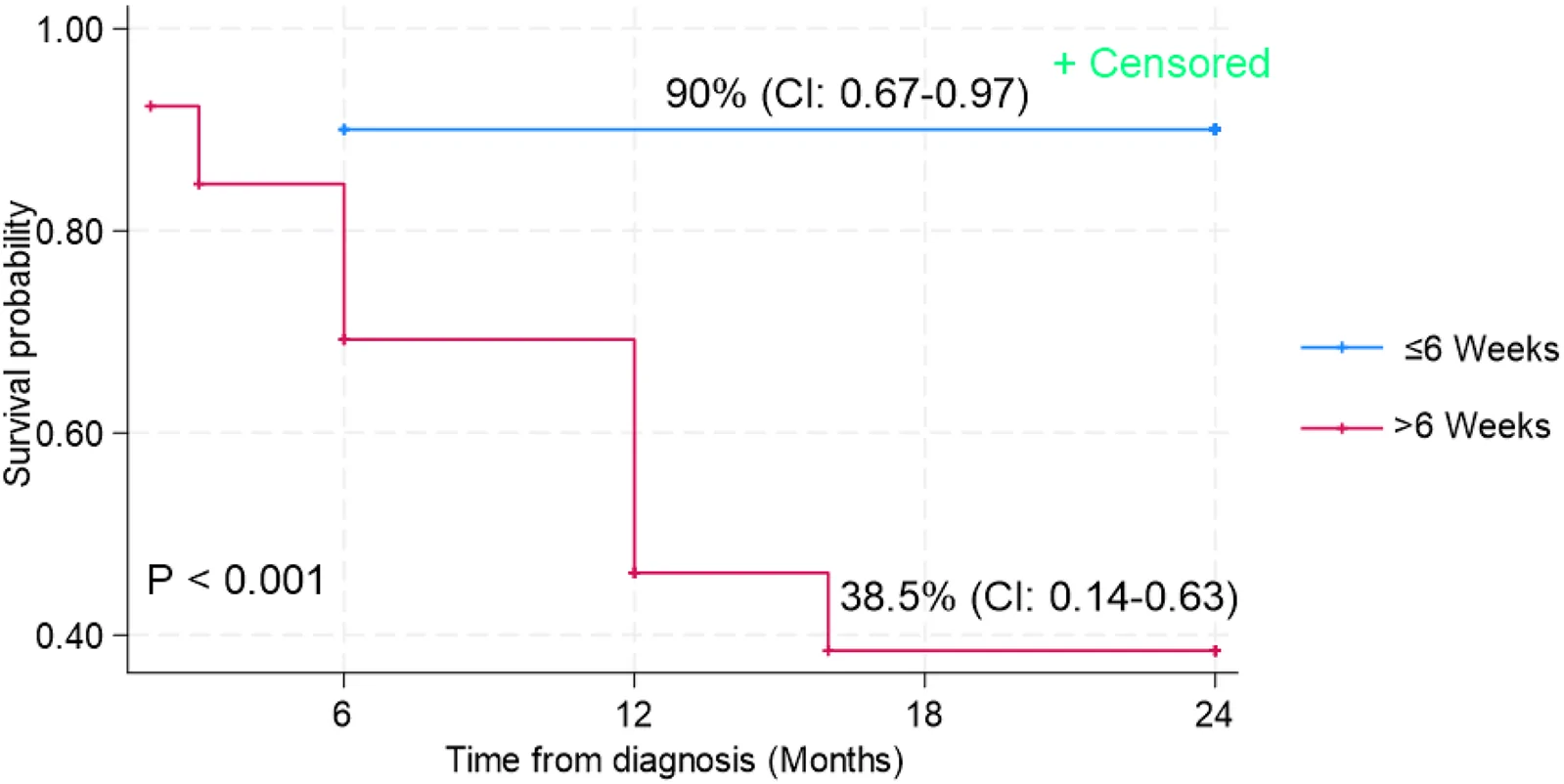
Kaplan-Meier survival estimates by time waiting for surgery.

Receipt of radiotherapy and hormonal therapy were associated with improved survival (Fig 6, Fig 7**).** However, these findings should be interpreted cautiously, as patients receiving these therapies were more likely to have better performance status and access to care, introducing potential confounding by indication.

**Fig 6:**
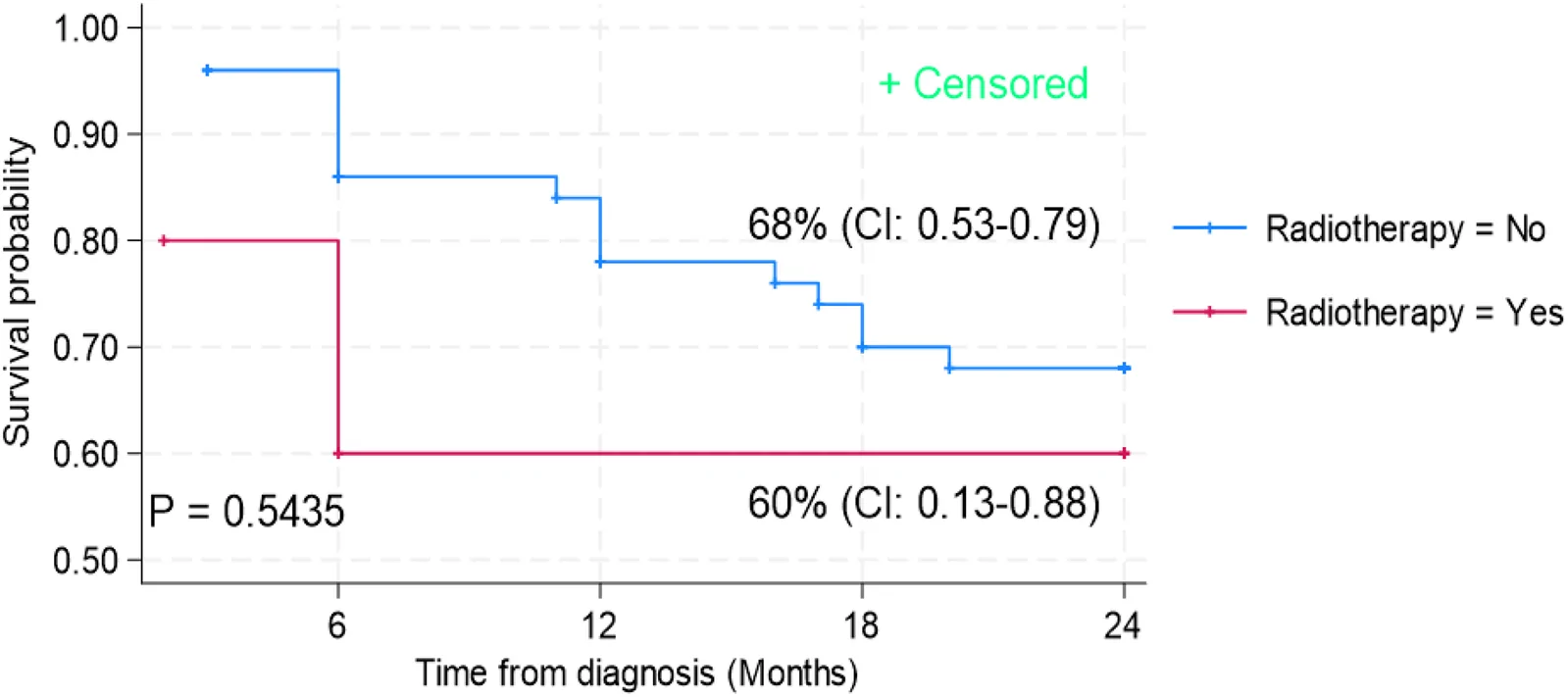
Kaplan-Meier survival estimates by radiotherapy.

**Fig 7:**
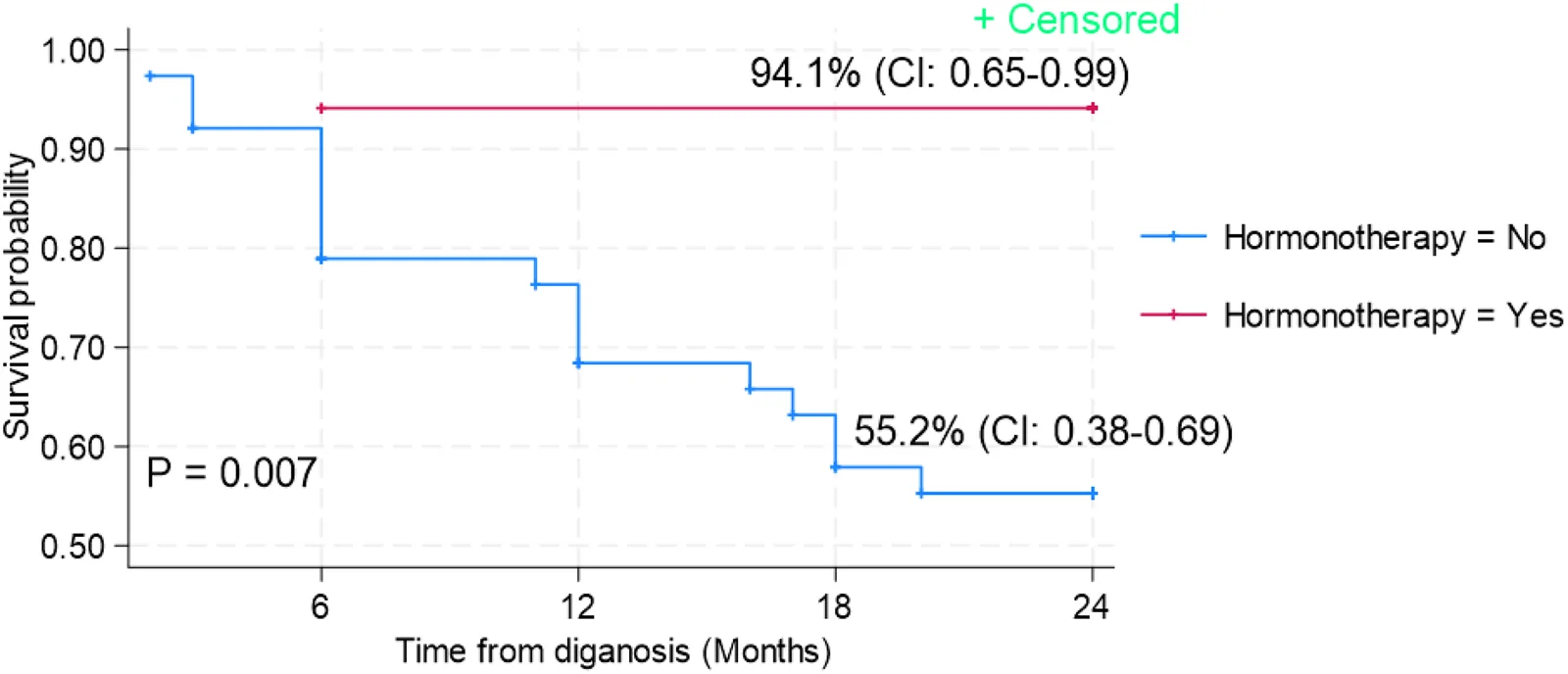
Kaplan-Meier survival estimates by hormonal therapy.

## Discussion

This study provides important insight into the clinicopathological characteristics and two-year survival of men with breast cancer treated at a tertiary referral center in Northern Tanzania. The findings confirm that male breast cancer predominantly affects older men and is frequently diagnosed at advanced stages, resulting in suboptimal survival outcomes [15,16].

The two-year overall survival rate of 67.3% is lower than that reported in high-income countries but comparable to other low- and middle-income settings [17]. This survival gap is likely driven by advanced stage at diagnosis, health-system delays, and limited access to radiotherapy and hormonal therapy rather than tumour biology alone.

A key contribution of this study is the demonstration that diagnostic and treatment delays independently predict mortality, consistent with previous evidence linking healthcare delays to advanced stage and worse survival [18–20].These findings highlight the critical role of health-system performance and suggest that improving turnaround times for pathology and surgical care could yield meaningful survival gains even without advanced therapies.

Radiotherapy and hormonal therapy were associated with improved survival, although utilization was low. Given the retrospective design, these associations should not be interpreted as causal; however, they underscore the potential survival benefit of expanding access to comprehensive cancer care services.

### Limitations

This study has several limitations, including small sample size, missing data for molecular subtyping, and limited follow-up duration. As a single-center tertiary referral study, the findings may overrepresent advanced disease and may not be fully generalizable to all settings in Tanzania.

## Conclusion

Male breast cancer in Northern Tanzania remains a clinically significant disease characterized by late presentation and suboptimal survival. Older age, advanced disease stage, and health-system delays are key determinants of poor outcomes. Interventions targeting early diagnosis, timely pathology reporting, and improved access to radiotherapy and hormonal therapy are essential to improve survival among men with breast cancer in low-resource settings.

## Supporting information

S1 Data Data set underlying the results. (XLSX)
