# Supplementary material for: Male breast cancer in Northern Tanzania: Clinicopathology and predictors of two-year survival (2016–2022)
Source: PLoS One. 2026 Nov 6;21(11):e0361615. doi: 10.1371/journal.pone.0361615 (PMC13742937; doi:10.1371/journal.pone.0361615)
Supplement: Supplementary file 1 [file pone.0361615.s003.docx]

**RESPONSE TO REVIEWERS’**

**Correspondence to Timon S. Emaely. Email: emaelytimon@gmail.com**

**KCMCU P.O. Box 2240, Moshi-Kilimanjaro, Tanzania, Phone +255 676 776 321**

**TITLE**: *Male Breast Cancer in Northern Tanzania: Clinicopathology and Predictors of Two-Year Survival (2016–2022).*

| SN | Reviewer No. 1 comments | Author Response | Page |
| --- | --- | --- | --- |
| 1 | The authors report in the abstract that 63.6% of male breast cancers were invasive ductal carcinoma. This is a surprisingly low fraction, given that invasive lobular carcinoma rarely if ever occurs in men. The authors correctly note that male breast tissue lacks lobules. The 63.6% statistic does not align with those in Table 3 reporting that 74.6% were IDC and 10.9% were Carcinoma NOS (all of which are likely ductal carcinoma. Please correct the data show in the abstract to align with those in tables. | *We thank the reviewer for this careful observation. We acknowledge that this was an error in the original manuscript. The percentage reported in the abstract (63.6%) did not correspond to the histological classification presented in Table 3. Upon review of our dataset, we found that cases classified as Carcinoma, NOS largely represent invasive ductal carcinomas that could not be further subtyped due to limited histopathological characterization. Therefore, in the revised manuscript, we have combined Invasive Ductal Carcinoma (IDC) and Carcinoma, NOS into a single category to more accurately reflect the histological distribution of male breast cancers. The abstract has been corrected accordingly to align with the data presented in Table 3. We appreciate the reviewer for identifying this inconsistency, which has improved the accuracy and clarity of the manuscript.* | 1, 6 and 15 |
| R1.2 | It is also surprising that 8/55 (15%) cases were classified as invasive lobular carcinoma. This is finding is sufficiently unusual to warrant verification. Did the authors seek an independent review of these 8 cases to verify they qualify as invasive lobular carcinoma and not ductal or mixed histologies? | *We thank the reviewer for raising this important point. We agree that invasive lobular carcinoma (ILC) is exceptionally uncommon in males because normal male breast tissue lacks well-developed lobules. In response to this concern, all eight cases initially classified as invasive lobular carcinoma were independently re-reviewed by a board-certified senior anatomical pathologist, who is also a co-author of this manuscript. The review included reassessment of the original hematoxylin and eosin (H&E) slides, and the diagnoses were confirmed based on the characteristic histomorphological features consistent with invasive lobular carcinoma. We have now clarified this verification process in the Methods section of the revised manuscript to assure readers that these uncommon diagnoses underwent independent expert review. We appreciate the reviewer for highlighting this important issue, which has strengthened the rigor and transparency of our study.* | 6 and 15 |
| R1.3 | The authors note that the proportion of male breast cancers is higher in sub-Saharan Africa (SSA) in comparison to high-income countries (HICs). The authors attribute this difference to factors related to the men diagnosed with disease (environmental exposures, genetic predisposition, and elevated oestrogen states associated with conditions such as hepatic schistosomiasis). This overlooks the primary difference comparing SSA with HICs, which relates to the lack of screening and early detection among women in SSA that leaves a large fraction of women undiagnosed in SSA compared to HICs. This is relevant from an epidemiological perspective but also from a policy perspective, because it suggests that awareness education, which they identify as a core need among men to reduce late-stage presentation, is equally or perhaps more important for women where the signifant majority of breast cancer cases and deaths occur. | *Thank you for this important comment. We agree that the higher reported proportion of male breast cancer in SSA may be influenced by limited screening, delayed diagnosis, and under-detection of male breast cancer rather than biological factors alone. The manuscript has been revised to acknowledge that awareness, early detection, and improved diagnostic access are critical interventions for both men and women.* | 2 |
| R1.4 | The authors correctly describe late-stage presentation as being a common obstacle for the management of male breast cancer. How different is this from presentation of female breast cancers in the same facility? This seems more like a common feature of male and female breast cancers and is not necessarily a distinguishing feature of male breast cancer. | *Thank you for this important comment. We agree that late-stage presentation is not unique to male breast cancer and also affects female breast cancer patients in our setting. The manuscript has been revised to present delayed presentation as a broader breast cancer management challenge related to awareness, health-seeking behaviour, and access to diagnostic services, while acknowledging potential additional delays among men due to disease rarity.* |  |
|  | Reviewer No. 2 comments |  |  |
| R2.1 | The authors describe the 2-year overall survival, however the describe in the methods that patients who died of causes outside breast cancer were excluded. So, is it really overall survival or disease specific survival? This needs to be clarified. | *We sincerely thank the reviewer for this careful observation. We acknowledge that this was an error in the original manuscript. The statement in the Methods section indicating that patients who died from causes unrelated to breast cancer were excluded was included in error and has now been removed in the revised manuscript. In fact, no patients were excluded based on cause of death. Therefore, the survival outcome reported in this study is overall survival (OS), defined as the time from diagnosis to death from any cause or the end of follow-up (censoring), rather than disease-specific survival. We have revised the Methods section accordingly to ensure consistency with the reported analyses and to avoid any ambiguity. We appreciate the reviewer for identifying this inconsistency, which has improved the accuracy and clarity of the manuscript.* | 3 |
| R2.2 | When the authors described delayed to surgery >6 weeks was associated with worse outcomes did they exclude the patients that received neoadjuvant chemotherapy? | *“Thank you for this important question. No, patients who received neoadjuvant chemotherapy were not excluded from this analysis. The definition of "delay to surgery" was based on the patient's treatment pathway. For patients who underwent upfront surgery, surgical delay was defined as an interval of more than 6 weeks from the date of diagnosis to surgery. For patients who received neoadjuvant chemotherapy, surgical delay was defined as an interval of more than 6 weeks from completion of neoadjuvant chemotherapy to surgery. Therefore, both groups were included, with the timing of surgery assessed relative to the appropriate clinical milestone for each treatment approach.* | 16 |
| R2.3 | Kaplan-Meier curves would be a very helpful adjunct in this paper | *Thank you for this observation. The Kaplan-Meier curves have been included in the revised manuscript, as suggested.* | 18-21 |
| R2.4 | It is unclear how authors handled missing data. For example, molecular subtype is unknown in 45.45% of cases and nodal status in 40%. Please clarify how missing data were handled in survival analyses and multivariable modeling. | *Missing data were handled using multiple imputation by chained equations (MICE) under the assumption that data were Missing at Random (MAR).* | 4 |
| R2.5 | Ethics approval reference number differs (PG 34/2024 in manuscript vs PG 40/2025) please clarify. Also was consent obtained from the participants? It is mentioned that it was waived and in another section that it was taken. Please clarify. | *Thank you for this important observation. To clarify, the correct ethical approval reference number is PG 34/2024. The alternative reference number that appeared in the manuscript was a typographical error. This has been corrected in the revised manuscript. In addition, a scanned copy of the original ethical approval document has been attached for* *verification and reference.*  *Regarding informed consent, the requirement for participant consent was waived by the Ethics Committee because this was a purely retrospective study that involved the review of existing patient records. There was no direct contact or communication between the research team and the study participants. The relevant statements in the manuscript have been revised to accurately reflect the Ethics Committee's waiver of informed consent and to align with standard reporting requirements.* | 5 |
| R2.6 | Radiotherapy was shown to be associated with improved survival. Which patients were included to see this benefit? Not all patients need to have RT after surgery so the patients who are not candidates should be excluded. | *Thank you for this insightful comment. We agree that not all patients with breast cancer require adjuvant radiotherapy after surgery, and including patients who were not candidates for radiotherapy could bias the observed association with survival. In response to this comment, we revised the analysis to include only patients who had an indication for postoperative radiotherapy according to the treatment protocol. Patients who were not candidates for radiotherapy were excluded from this specific analysis. The corresponding description in the Methods and Results sections has been revised accordingly to clarify the study population used to evaluate the effect of radiotherapy on survival.* | 17 |
|  | Editor’s comments |  |  |
| Editor 1 | The ethical approval number listed in the manuscript (Ref. No. PG 34/2024) and submission metadata (Ref. No. PG 40/2025) does not match. Please ensure that all approval numbers are correct. | *Thank you for identifying this discrepancy. We confirm that the correct ethical approval reference number is* ***PG 34/2024****. The alternative reference number (****PG 40/2025****) was a typographical error and has been corrected. The ethical approval details are now consistent throughout the manuscript, and a scanned copy of the original ethical approval document has been provided for verification.* | 5 |
| Editor 2 | We note that the grant information you provided in the ‘Funding Information’ and ‘Financial Disclosure’ sections do not match.  When you resubmit, please ensure that you provide the correct grant numbers for the awards you received for your study in the ‘Funding Information’ section. | *We thank the Editor for highlighting this discrepancy. We acknowledge that the grant information provided in the original submission was inconsistent. We have carefully reviewed and corrected the funding statement in the revised manuscript to ensure that the appropriate grant information is accurately reported. The revised funding information now includes the following statement: The project on which this publication is based was in part funded by the German Federal Ministry of Research, Technology and Space 01KA2220B under NORA project. This research was funded in part by Science for Africa Foundation with support from Wellcome Trust and the UK Foreign, Commonwealth & Development Office and is part of the EDCPT2 programme supported by the European Union.* |  |
| Editor 3 | Please state what role the funders took in the study. If the funders had no role, please state: "The funders had no role in study design, data collection and analysis, decision to publish, or preparation of the manuscript."  If this statement is not correct you must amend it as needed.  Please include this amended Role of Funder statement in your cover letter; we will change the online submission form on your behalf. | *The funders had no role in study design, data collection and analysis, decision to publish, or preparation of the manuscript."* |  |
| Editor 4 | Please note that funding information should not appear in any section or other areas of your manuscript. We will only publish funding information present in the Funding Statement section of the online submission form. Please remove any funding-related text from the manuscript. | *Thank you for this comment. Funding information from the manuscript has been removed*. | 8 |
| Editor 5 | Your ethics statement should only appear in the Methods section of your manuscript. If your ethics statement is written in any section besides the Methods, please move it to the Methods section and delete it from any other section. Please ensure that your ethics statement is included in your manuscript, as the ethics statement entered into the online submission form will not be published alongside your manuscript. | *Thank you for this comment. We have revised the manuscript accordingly by ensuring that the ethics statement appears only in the Methods section. It has been removed from all other sections, and the complete ethics statement is now included in the manuscript for publication.* | 5 |
| Editor 6 | We note you have included a table to which you do not refer in the text of your manuscript. Please ensure that you refer to Tables 1 to 5 in your text; if accepted, production will need this reference to link the reader to the Table. | *Thank you for pointing this out. We have revised the manuscript to ensure that all tables (Tables 1–5) are appropriately cited in the text at the relevant locations.* |  |
