# Supplementary material for: Male breast cancer in Northern Tanzania: Clinicopathology and predictors of two-year survival (2016–2022)
Source: PLoS One. 2026 Nov 6;21(11):e0361615. doi: 10.1371/journal.pone.0361615 (PMC13742937; doi:10.1371/journal.pone.0361615)
Supplement: Peer review history

# Peer review: Decision Letter 0

**DOI:** 10.1371/journal.pone.0361615.r001

**Authors:** Helen Howard

1 Jun 2026

PONE-D-26-01223

Male Breast Cancer in Northern Tanzania: Clinicopathology and Predictors of Two-Year Survival (2016–2022)

PLOS One

Dear Dr. Emaely,

Thank you for submitting your manuscript to PLOS ONE. After careful consideration, we feel that it has merit but does not fully meet PLOS ONE’s publication criteria as it currently stands. Therefore, we invite you to submit a revised version of the manuscript that addresses the points raised during the review process.

The manuscript has been evaluated by two reviewers, and their comments are available below.

The reviewers have raised a number of concerns that need attention. In particular, they request additional information on the methodological and statistical aspects of the study.

Could you please revise the manuscript to carefully address the concerns raised?

Please submit your revised manuscript by Jul 14 2026 11:59PM. If you will need more time than this to complete your revisions, please reply to this message or contact the journal office at plosone@plos.org. When you're ready to submit your revision, log on to https://www.editorialmanager.com/pone/ and select the 'Submissions Needing Revision' folder to locate your manuscript file.

Please include the following items when submitting your revised manuscript:

- A letter that responds to each point raised by the academic editor and reviewer(s). You should upload this letter as a separate file labeled 'Response to Reviewers'.
- A marked-up copy of your manuscript that highlights changes made to the original version. You should upload this as a separate file labeled 'Revised Manuscript with Track Changes'.
- An unmarked version of your revised paper without tracked changes. You should upload this as a separate file labeled 'Manuscript'

If you would like to make changes to your financial disclosure, please include your updated statement in your cover letter. Guidelines for resubmitting your figure files are available below the reviewer comments at the end of this letter.

If applicable, we recommend that you deposit your laboratory protocols in protocols.io to enhance the reproducibility of your results. Protocols.io assigns your protocol its own identifier (DOI) so that it can be cited independently in the future. For instructions see: https://journals.plos.org/plosone/s/submission-guidelines#loc-laboratory-protocols. Additionally, PLOS ONE offers an option for publishing peer-reviewed Lab Protocol articles, which describe protocols hosted on protocols.io. Read more information on sharing protocols at https://plos.org/protocols?utm_medium=editorial-email&utm_source=authorletters&utm_campaign=protocols.

As the corresponding author, your ORCID iD is verified in the submission system and will appear in the published article. PLOS supports the use of ORCID, and we encourage all coauthors to register for an ORCID iD and use it as well. Please encourage your coauthors to verify their ORCID iD within the submission system before final acceptance, as unverified ORCID iDs will not appear in the published article. *Only*  the individual author can complete the verification step; PLOS staff *cannot*  verify ORCID iDs on behalf of authors.

We look forward to receiving your revised manuscript.

Kind regards,

Helen Howard

Staff Editor

PLOS One

**Journal Requirements:**

When submitting your revision, we need you to address these additional requirements.

1. Please ensure that your manuscript meets PLOS ONE's style requirements, including those for file naming. The PLOS ONE style templates can be found at

https://journals.plos.org/plosone/s/file?id=wjVg/PLOSOne_formatting_sample_main_body.pdf and

https://journals.plos.org/plosone/s/file?id=ba62/PLOSOne_formatting_sample_title_authors_affiliations.pdf

2. The ethical approval number listed in the manuscript  (Ref. No. PG 34/2024) and submission metadata (Ref. No. PG 40/2025) does not match. Please ensure that all approval numbers are correct.

3. We note that the grant information you provided in the ‘Funding Information’ and ‘Financial Disclosure’ sections do not match.

When you resubmit, please ensure that you provide the correct grant numbers for the awards you received for your study in the ‘Funding Information’ section.

4. Thank you for stating the following financial disclosure:

“This work was supported by  The University of Alabama (UAB), Kilimanjaro Clinical Research Institute (KCRI), NORA Consortium, funded by the German Federal Ministry of Research, Technology and Space 01KA2220 and REACCT-CAN funded by Science for Africa Foundation to the Developing Excellence in Leadership, Training and Science in Africa (DELTAS Africa) program [Del-22-008] with support from Wellcome Trust and the UK Foreign, Commonwealth & Development Office and is part of the EDCPT2 programme supported by the European Union and Global Health Working Group, Martin-Luther-University Halle-Wittenberg (Grant numbers [81295996]. The authors are responsible for the study.”

Please state what role the funders took in the study.  If the funders had no role, please state: "The funders had no role in study design, data collection and analysis, decision to publish, or preparation of the manuscript."

If this statement is not correct you must amend it as needed.

Please include this amended Role of Funder statement in your cover letter; we will change the online submission form on your behalf.

5. Please note that funding information should not appear in any section or other areas of your manuscript. We will only publish funding information present in the Funding Statement section of the online submission form. Please remove any funding-related text from the manuscript.

6. We note that your Data Availability Statement is currently as follows:

“All relevant data are within the manuscript and its Supporting Information files.”

Please confirm at this time whether or not your submission contains all raw data required to replicate the results of your study. Authors must share the “minimal data set” for their submission. PLOS defines the minimal data set to consist of the data required to replicate all study findings reported in the article, as well as related metadata and methods (https://journals.plos.org/plosone/s/data-availability#loc-minimal-data-set-definition).

For example, authors should submit the following data:

- The values behind the means, standard deviations and other measures reported;

- The values used to build graphs;

- The points extracted from images for analysis.

Authors do not need to submit their entire data set if only a portion of the data was used in the reported study.

If your submission does not contain these data, please either upload them as Supporting Information files or deposit them to a stable, public repository and provide us with the relevant URLs, DOIs, or accession numbers. For a list of recommended repositories, please see https://journals.plos.org/plosone/s/recommended-repositories.

If there are ethical or legal restrictions on sharing a de-identified data set, please explain them in detail (e.g., data contain potentially sensitive information, data are owned by a third-party organization, etc.) and who has imposed them (e.g., an ethics committee). Please also provide contact information for a data access committee, ethics committee, or other institutional body to which data requests may be sent. If data are owned by a third party, please indicate how others may request data access.

7. Your ethics statement should only appear in the Methods section of your manuscript. If your ethics statement is written in any section besides the Methods, please move it to the Methods section and delete it from any other section. Please ensure that your ethics statement is included in your manuscript, as the ethics statement entered into the online submission form will not be published alongside your manuscript.

8. We note you have included a table to which you do not refer in the text of your manuscript. Please ensure that you refer to Tables 1 to 5 in your text; if accepted, production will need this reference to link the reader to the Table.

9. If the reviewer comments include a recommendation to cite specific previously published works, please review and evaluate these publications to determine whether they are relevant and should be cited. There is no requirement to cite these works unless the editor has indicated otherwise.

[Note: HTML markup is below. Please do not edit.]

Reviewers' comments:

Reviewer's Responses to Questions

**Comments to the Author**

1. Is the manuscript technically sound, and do the data support the conclusions?

The manuscript must describe a technically sound piece of scientific research with data that supports the conclusions. Experiments must have been conducted rigorously, with appropriate controls, replication, and sample sizes. The conclusions must be drawn appropriately based on the data presented.

Reviewer #1: Partly

Reviewer #2: Partly

**********

2. Has the statistical analysis been performed appropriately and rigorously?

Reviewer #1: Yes

Reviewer #2: No

**********

3. Have the authors made all data underlying the findings in their manuscript fully available?

The PLOS Data policy requires authors to make all data underlying the findings described in their manuscript fully available without restriction, with rare exception (please refer to the Data Availability Statement in the manuscript PDF file). The data should be provided as part of the manuscript or its supporting information, or deposited to a public repository. For example, in addition to summary statistics, the data points behind means, medians and variance measures should be available. If there are restrictions on publicly sharing data—e.g. participant privacy or use of data from a third party—those must be specified.

Reviewer #1: Yes

Reviewer #2: Yes

**********

4. Is the manuscript presented in an intelligible fashion and written in standard English?

PLOS ONE does not copyedit accepted manuscripts, so the language in submitted articles must be clear, correct, and unambiguous. Any typographical or grammatical errors should be corrected at revision, so please note any specific errors here.

Reviewer #1: Yes

Reviewer #2: Yes

**********

5. Review Comments to the Author

Please use the space provided to explain your answers to the questions above. You may also include additional comments for the author, including concerns about dual publication, research ethics, or publication ethics. (Please upload your review as an attachment if it exceeds 20,000 characters)

Reviewer #1: The authors report in the abstract that 63.6% of male breast cancers were invasive ductal carcinoma. This is a surprisingly low fraction, given that invasive lobular carcinoma rarely if ever occurs in men. The authors correctly note that male breast tissue lacks lobules. The 63.6% statistic does not align with those in Table 3 reporting that 74.6% were IDC and 10.9% were Carcinoma NOS (all of which are likely ductal carcinoma. Please correct the data show in the abstract to align with those in tables.

It is also surprising that 8/55 (15%) cases were classified as invasive lobular carcinoma. This is finding is sufficiently unusual to warrant verification. Did the authors seek an independent review of these 8 cases to verify they qualify as invasive lobular carcinoma and not ductal or mixed histologies?

The authors note that the proportion of male breast cancers is higher in sub-Saharan Africa (SSA) in comparison to high-income countries (HICs). The authors attribute this diffence to factors related to the men diagnosed with disease (environmental exposures, genetic predisposition, and elevated oestrogen states associated with conditions such as hepatic schistosomiasis). This overlooks the primary difference comparing SSA with HICs, which relates to the lack of screening and early detection among women in SSA that leaves a large fraction of women undiagnosed in SSA compared to HICs. This is relevant from an epidemiological perspective but also from a policy perspective, because it suggests that awareness education, which they identify as a core need among men to reduce late-stage presentation, is equally or perhaps more important for women where the signifant majority of breast cancer cases and deaths occur.

The authors correctly describe late-stage presentation as being a common obstacle for the management of male breast cancer. How different is this from presentation of female breast cancers in the same facility? This seems more like a common feature of male and female breast cancers and is not necessarily a distinguishing feature of male breast cancer.

Reviewer #2: 1. The authors describe the 2-year overall survival, however the describe in the methods that patients who died of causes outside breast cancer were excluded. So is it really overall survival or disease specific survival? This needs to be clarified.

2. When the authors described delayed to surgery >6 weeks was associated with worse outcomes did they exclude the patients that received neoadjuvant chemotherapy?

3. Kaplan-Meier curves would be a very helpful adjunct in this paper

4. It is unclear how authors handled missing data. For example molecular subtype is unknown in 45.45% of cases and nodal status in 40%. Please clarify how missing data were handled in survival analyses and multivariable modeling.

5. Ethics approval reference number differs (PG 34/2024 in manuscript vs PG 40/2025) please clarify. Also was consent obtained from the participants? It is mentioned that it was waived and in another section that it was taken. Please clarify.

6. Radiotherapy was shown to be associated with improved survival. Which patients were included to see this benefit? Not all patients need to have RT after surgery so the patients who are not candidates should be excluded.

**********

6. PLOS authors have the option to publish the peer review history of their article (what does this mean?). If published, this will include your full peer review and any attached files.

If you choose “no”, your identity will remain anonymous but your review may still be made public.

**Do you want your identity to be public for this peer review?**  For information about this choice, including consent withdrawal, please see our Privacy Policy.

Reviewer #1: **Yes:**  Benjamin O Anderson

Reviewer #2: No

**********

[NOTE: If reviewer comments were submitted as an attachment file, they will be attached to this email and accessible via the submission site. Please log into your account, locate the manuscript record, and check for the action link "View Attachments". If this link does not appear, there are no attachment files.]

To ensure your figures meet our technical requirements, please review our figure guidelines: https://journals.plos.org/plosone/s/figures

You may also use PLOS’s free figure tool, NAAS, to help you prepare publication quality figures: https://journals.plos.org/plosone/s/figures#loc-tools-for-figure-preparation.

NAAS will assess whether your figures meet our technical requirements by comparing each figure against our figure specifications.

# Author comment: Author response to Decision Letter 1

**DOI:** 10.1371/journal.pone.0361615.r002

19 Sep 2026

Reviewer No. 1 comments

1. The authors report in the abstract that 63.6% of male breast cancers were invasive ductal carcinoma. This is a surprisingly low fraction, given that invasive lobular carcinoma rarely if ever occurs in men. The authors correctly note that male breast tissue lacks lobules. The 63.6% statistic does not align with those in Table 3 reporting that 74.6% were IDC and 10.9% were Carcinoma NOS (all of which are likely ductal carcinoma. Please correct the data show in the abstract to align with those in tables.

RESPONSE

We thank the reviewer for this careful observation. We acknowledge that this was an error in the original manuscript. The percentage reported in the abstract (63.6%) did not correspond to the histological classification presented in Table 3. Upon review of our dataset, we found that cases classified as Carcinoma, NOS largely represent invasive ductal carcinomas that could not be further subtyped due to limited histopathological characterization. Therefore, in the revised manuscript, we have combined Invasive Ductal Carcinoma (IDC) and Carcinoma, NOS into a single category to more accurately reflect the histological distribution of male breast cancers. The abstract has been corrected accordingly to align with the data presented in Table 3. We appreciate the reviewer for identifying this inconsistency, which has improved the accuracy and clarity of the manuscript.

LOCATION; Page 1, 6 and 15

2. It is also surprising that 8/55 (15%) cases were classified as invasive lobular carcinoma. This is finding is sufficiently unusual to warrant verification. Did the authors seek an independent review of these 8 cases to verify they qualify as invasive lobular carcinoma and not ductal or mixed histologies?

RESPONSE

We thank the reviewer for raising this important point. We agree that invasive lobular carcinoma (ILC) is exceptionally uncommon in males because normal male breast tissue lacks well-developed lobules. In response to this concern, all eight cases initially classified as invasive lobular carcinoma were independently re-reviewed by a board-certified senior anatomical pathologist, who is also a co-author of this manuscript. The review included reassessment of the original hematoxylin and eosin (H&E) slides, and the diagnoses were confirmed based on the characteristic histomorphological features consistent with invasive lobular carcinoma. We have now clarified this verification process in the Methods section of the revised manuscript to assure readers that these uncommon diagnoses underwent independent expert review. We appreciate the reviewer for highlighting this important issue, which has strengthened the rigor and transparency of our study.

LOCATION; Page 6 and 15

3. The authors note that the proportion of male breast cancers is higher in sub-Saharan Africa (SSA) in comparison to high-income countries (HICs). The authors attribute this difference to factors related to the men diagnosed with disease (environmental exposures, genetic predisposition, and elevated oestrogen states associated with conditions such as hepatic schistosomiasis). This overlooks the primary difference comparing SSA with HICs, which relates to the lack of screening and early detection among women in SSA that leaves a large fraction of women undiagnosed in SSA compared to HICs. This is relevant from an epidemiological perspective but also from a policy perspective, because it suggests that awareness education, which they identify as a core need among men to reduce late-stage presentation, is equally or perhaps more important for women where the signifant majority of breast cancer cases and deaths occur.

RESPONSE

Thank you for this important comment. We agree that the higher reported proportion of male breast cancer in SSA may be influenced by limited screening, delayed diagnosis, and under-detection of male breast cancer rather than biological factors alone. The manuscript has been revised to acknowledge that awareness, early detection, and improved diagnostic access are critical interventions for both men and women.

LOCATION; Page 2

4. The authors correctly describe late-stage presentation as being a common obstacle for the management of male breast cancer. How different is this from presentation of female breast cancers in the same facility? This seems more like a common feature of male and female breast cancers and is not necessarily a distinguishing feature of male breast cancer.

RESPONSE; Thank you for this important comment. We agree that late-stage presentation is not unique to male breast cancer and also affects female breast cancer patients in our setting. The manuscript has been revised to present delayed presentation as a broader breast cancer management challenge related to awareness, health-seeking behaviour, and access to diagnostic services, while acknowledging potential additional delays among men due to disease rarity

Reviewer No. 2 comments

1. The authors describe the 2-year overall survival, however the describe in the methods that patients who died of causes outside breast cancer were excluded. So, is it really overall survival or disease specific survival? This needs to be clarified.

RESPONSE; We sincerely thank the reviewer for this careful observation. We acknowledge that this was an error in the original manuscript. The statement in the Methods section indicating that patients who died from causes unrelated to breast cancer were excluded was included in error and has now been removed in the revised manuscript. In fact, no patients were excluded based on cause of death. Therefore, the survival outcome reported in this study is overall survival (OS), defined as the time from diagnosis to death from any cause or the end of follow-up (censoring), rather than disease-specific survival. We have revised the Methods section accordingly to ensure consistency with the reported analyses and to avoid any ambiguity. We appreciate the reviewer for identifying this inconsistency, which has improved the accuracy and clarity of the manuscript.

LOCATION; Page 3

2. When the authors described delayed to surgery >6 weeks was associated with worse outcomes did they exclude the patients that received neoadjuvant chemotherapy

RESPONSE; “Thank you for this important question. No, patients who received neoadjuvant chemotherapy were not excluded from this analysis. The definition of "delay to surgery" was based on the patient's treatment pathway. For patients who underwent upfront surgery, surgical delay was defined as an interval of more than 6 weeks from the date of diagnosis to surgery. For patients who received neoadjuvant chemotherapy, surgical delay was defined as an interval of more than 6 weeks from completion of neoadjuvant chemotherapy to surgery. Therefore, both groups were included, with the timing of surgery assessed relative to the appropriate clinical milestone for each treatment approach.

LOCATION; Page 16

3. Kaplan-Meier curves would be a very helpful adjunct in this paper

RESPONSE;Thank you for this observation. The Kaplan-Meier curves have been included in the revised manuscript, as suggested.

LOCATION; Page 18-21

4. It is unclear how authors handled missing data. For example, molecular subtype is unknown in 45.45% of cases and nodal status in 40%. Please clarify how missing data were handled in survival analyses and multivariable modeling.

RESPONSE; Missing data were handled using multiple imputation by chained equations (MICE) under the assumption that data were Missing at Random (MAR).

LOCATION; Page 4

5. Ethics approval reference number differs (PG 34/2024 in manuscript vs PG 40/2025) please clarify. Also was consent obtained from the participants? It is mentioned that it was waived and in another section that it was taken. Please clarify

RESPONSE; Thank you for this important observation. To clarify, the correct ethical approval reference number is PG 34/2024. The alternative reference number that appeared in the manuscript was a typographical error. This has been corrected in the revised manuscript. In addition, a scanned copy of the original ethical approval document has been attached for verification and reference.

Regarding informed consent, the requirement for participant consent was waived by the Ethics Committee because this was a purely retrospective study that involved the review of existing patient records. There was no direct contact or communication between the research team and the study participants. The relevant statements in the manuscript have been revised to accurately reflect the Ethics Committee's waiver of informed consent and to align with standard reporting requirements.

LOCATION; Page 5

6. Radiotherapy was shown to be associated with improved survival. Which patients were included to see this benefit? Not all patients need to have RT after surgery so the patients who are not candidates should be excluded.

RESPONSE; Thank you for this insightful comment. We agree that not all patients with breast cancer require adjuvant radiotherapy after surgery, and including patients who were not candidates for radiotherapy could bias the observed association with survival. In response to this comment, we revised the analysis to include only patients who had an indication for postoperative radiotherapy according to the treatment protocol. Patients who were not candidates for radiotherapy were excluded from this specific analysis. The corresponding description in the Methods and Results sections has been revised accordingly to clarify the study population used to evaluate the effect of radiotherapy on survival.

LOCATION; Page 17

Editor’s comments

1. The ethical approval number listed in the manuscript (Ref. No. PG 34/2024) and submission metadata (Ref. No. PG 40/2025) does not match. Please ensure that all approval numbers are correct

RESPONSE; Thank you for identifying this discrepancy. We confirm that the correct ethical approval reference number is PG 34/2024. The alternative reference number (PG 40/2025) was a typographical error and has been corrected. The ethical approval details are now consistent throughout the manuscript, and a scanned copy of the original ethical approval document has been provided for verification.

LOCATION; Page 5

2. We note that the grant information you provided in the ‘Funding Information’ and ‘Financial Disclosure’ sections do not match.

When you resubmit, please ensure that you provide the correct grant numbers for the awards you received for your study in the ‘Funding Information’ section.

RESPONSE; We thank the Editor for highlighting this discrepancy. We acknowledge that the grant information provided in the original submission was inconsistent. We have carefully reviewed and corrected the funding statement in the revised manuscript to ensure that the appropriate grant information is accurately reported. The revised funding information now includes the following statement: The project on which this publication is based was in part funded by the German Federal Ministry of Research, Technology and Space 01KA2220B under NORA project. This research was funded in part by Science for Africa Foundation with support from Wellcome Trust and the UK Foreign, Commonwealth & Development Office and is part of the EDCPT2 programme supported by the European Union.

3.Please state what role the funders took in the study. If the funders had no role, please state: "The funders had no role in study design, data collection and analysis, decision to publish, or preparation of the manuscript."

If this statement is not correct you must amend it as needed.

Please include this amended Role of Funder statement in your cover letter; we will change the online submission form on your behalf.

RESPONSE; The funders had no role in study design, data collection and analysis, decision to publish, or preparation of the manuscript."

4. Please note that funding information should not appear in any section or other areas of your manuscript. We will only publish funding information present in the Funding Statement section of the online submission form. Please remove any funding-related text from the manuscript.

RESPONSE; Thank you for this comment. Funding information from the manuscript has been removed.

LOCATION; Page 8

5. Your ethics statement should only appear in the Methods section of your manuscript. If your ethics statement is written in any section besides the Methods, please move it to the Methods section and delete it from any other section. Please ensure that your ethics statement is included in your manuscript, as the ethics statement entered into the online submission form will not be published alongside your manuscript.

RESPONSE; Thank you for this comment. We have revised the manuscript accordingly by ensuring that the ethics statement appears only in the Methods section. It has been removed from all other sections, and the complete ethics statement is now included in the manuscript for publication.

LOCATION; Page 5

We note you have included a table to which you do not refer in the text of your manuscript. Please ensure that you refer to Tables 1 to 5 in your text; if accepted, production will need this reference to link the reader to the Table.

RESPONSE; Thank you for pointing this out. We have revised the manuscript to ensure that all tables (Tables 1–5) are appropriately cited in the text at the relevant locations.

6.

Attachment Submitted filename: RESPONSE TO REVIEWERS.docx

# Peer review: Decision Letter 1

**DOI:** 10.1371/journal.pone.0361615.r003

**Authors:** Madhura Kulkarni

13 Oct 2026

Male Breast Cancer in Northern Tanzania: Clinicopathology and Predictors of Two-Year Survival (2016–2022)

PONE-D-26-01223R1

Dear Dr. Emaely,

We’re pleased to inform you that your manuscript has been judged scientifically suitable for publication and will be formally accepted for publication once it meets all outstanding technical requirements.

Within one week, you’ll receive an e-mail detailing the required amendments. When these have been addressed, you’ll receive a formal acceptance letter and your manuscript will be scheduled for publication.

An invoice will be generated when your article is formally accepted. Please note, if your institution has a publishing partnership with PLOS and your article meets the relevant criteria, all or part of your publication costs will be covered. Please make sure your user information is up-to-date by logging into Editorial Manager at Editorial Manager® and clicking the ‘Update My Information' link at the top of the page. For questions related to billing, please contact billing support.

If your institution or institutions have a press office, please notify them about your upcoming paper to help maximize its impact. If they’ll be preparing press materials, please inform our press team as soon as possible -- no later than 48 hours after receiving the formal acceptance. Your manuscript will remain under strict press embargo until 2 pm Eastern Time on the date of publication. For more information, please contact onepress@plos.org.

Kind regards,

Madhura Kulkarni

Academic Editor

PLOS One

Additional Editor Comments (optional):

Reviewers' comments:

Reviewer's Responses to Questions

**Comments to the Author**

Reviewer #1: All comments have been addressed

**********

2. Is the manuscript technically sound, and do the data support the conclusions?

Reviewer #1: Yes

**********

3. Has the statistical analysis been performed appropriately and rigorously?

Reviewer #1: Yes

**********

4. Have the authors made all data underlying the findings in their manuscript fully available?

The PLOS Data policy

Reviewer #1: Yes

**********

5. Is the manuscript presented in an intelligible fashion and written in standard English?

Reviewer #1: Yes

**********

Reviewer #1: The authors have fully responded to my comments from the first review. The data additions and clarifications have improved the manuscript clarity and accuracy.

**********

what does this mean?). If published, this will include your full peer review and any attached files.

If you choose “no”, your identity will remain anonymous but your review may still be made public.

**Do you want your identity to be public for this peer review?**  For information about this choice, including consent withdrawal, please see our Privacy Policy

Reviewer #1: No

**********

# Editor report: Acceptance letter

**DOI:** 10.1371/journal.pone.0361615.r004

**Authors:** Madhura Kulkarni

PONE-D-26-01223R1

PLOS One

Dear Dr. Emaely,

I'm pleased to inform you that your manuscript has been deemed suitable for publication in PLOS One. Congratulations! Your manuscript is now being handed over to our production team.

At this stage, our production department will prepare your paper for publication. This includes ensuring the following:

* All references, tables, and figures are properly cited

* All relevant supporting information is included in the manuscript submission,

* There are no issues that prevent the paper from being properly typeset

You will receive further instructions from the production team, including instructions on how to review your proof when it is ready. Please keep in mind that we are working through a large volume of accepted articles, so please give us a few days to review your paper and let you know the next and final steps.

Lastly, if your institution or institutions have a press office, please let them know about your upcoming paper now to help maximize its impact. If they'll be preparing press materials, please inform our press team within the next 48 hours. Your manuscript will remain under strict press embargo until 2 pm Eastern Time on the date of publication. For more information, please contact onepress@plos.org.

You will receive an invoice from PLOS for your publication fee after your manuscript has reached the completed accept phase. If you receive an email requesting payment before acceptance or for any other service, this may be a phishing scheme. Learn how to identify phishing emails and protect your accounts at https://explore.plos.org/phishing.

If we can help with anything else, please email us at customercare@plos.org.

Thank you for submitting your work to PLOS One and supporting open access.

Kind regards,

PLOS One Editorial Office Staff

on behalf of

Dr. Madhura Kulkarni

Academic Editor

PLOS One
